# COVID-19 in Africa: an ovarian victory?

**DOI:** 10.1186/s13048-021-00820-1

**Published:** 2021-05-21

**Authors:** Osman A. Dufailu, Afrakoma Afriyie-Asante, Bernard Gyan, David Adu Kwabena, Helena Yeboah, Frank Ntiakoh, Meshach Asare-Werehene

**Affiliations:** 1 Department of Microbiology, Faculty of Biosciences, University for Development Studies, Box 1882, Nyankpala Campus, Tamale, Ghana; 2 Department of Biochemistry, Microbiology and Immunology, Faculty of Medicine, University of Ottawa, Ottawa, Ontario K1H 8M5 Canada; 3 Department of Medical Diagnostics, College of Health and Well-Being, Kintampo, Ghana; 4 School of Allied Health Sciences, University for Development Studies, Tamale, Ghana; 5 School of International Development and Global Studies, University of Ottawa, Ottawa, Ontario Canada; 6 Department of Medical Laboratory, Effia-Nkwanta Regional Hospital, Sekondi, Western Region Ghana; 7 Departments of Cellular and Molecular Medicine and Obstetrics and Gynecology, University of Ottawa, Ottawa, Ontario K1H 8M5 Canada; 8 Chronic Disease Program, Ottawa Hospital Research Institute, The Ottawa Hospital, Ottawa, Ontario K1H 8L6 Canada

**Keywords:** COVID-19, SARS-CoV-2, Africa, Ovary, Mortality rate, Estrogen, Pro-inflammation cytokine

## Abstract

Coronavirus disease 2019 (COVID-19) caused by Severe Acute Respiratory Syndrome Coronavirus 2 (SARS-CoV-2) mainly attacks the respiratory system and is characterized by pneumonia, cytokine storm, coagulation disorders and severe immune downregulation. Although public health experts predicted worst outcomes in Africa, the incidence, hospitalization and mortality rates have been lower in Africa compared to other continents. Interestingly, lower incidence and mortality rates have been observed in women from Africa compared to their cohorts from other continents. Also, in the US non-Hispanic Black females have lower COVID-19 and death rates compared to their white counterparts. Its unclear why this significant difference exists; however, the ovarian function, genetics and immunological statuses could play a major role. Women of African descent have elevated levels of estrogen compared with Caucasians hence we anticipate that estrogen might offer some protection against the SARS-CoV-2 infections. The racial differences in lifestyle, age and inaccessibility to contraceptive usage might also play a role. Here, we provide insight on how the high levels of estrogen in African women might contribute to the lower cases and fatalities in Africa. Specifically, estrogen might offer protection against COVID-19 by suppressing hyper-production of cytokines, promoting anti-inflammatory cytokines, stimulating antibody production and suppressing endoplasmic reticulum (ER) stress. This will as well provide useful information on how future pandemics could be managed using Africa as a case study.

## Background of coronavirus disease 2019 (COVID-19)

COVID-19 is caused by Severe Acute Respiratory Syndrome Coronavirus 2 (SARS-CoV-2). This novel virus was first identified in Wuhan, China to be causing an atypical form of pneumonia and has since spread to most parts of the world due to the fluidity of the human populace [1, 2]. The disease was declared a pandemic by the world health organization (WHO) in March, 2020. SARS-CoV-2 is mainly transmitted via respiratory droplets and aerosols although other studies have suggested potential fecal and airborne transmissions [3, 4]. COVID-19 is characterized by hyper-production of inflammatory cytokines (cytokine storm), down-regulated immune system, coagulation disorders, multiple organ dysfunctions (MOD) and in severe cases neurological problems [5,8]. Currently, the gold standard for the diagnosis of COVID-19 is real time reverse transcriptase polymerase chain reaction (rRT-PCR) assays of respiratory samples obtained from nasopharyngeal and oropharyngeal swabs although antibody detection in the serum could indicate exposure. Other samples that could be used are obtained from bronchoalveolar lavage, serum and sputum [9]. The treatment of COVID-19 is mainly supportive as there is no world health organization (WHO) approved protocol for treatment. Clinical management currently include the use of hydroxychloroquine-azithromycin combination, analgesics, corticosteroids, remdesivir, ritonavir, rintatolimvir and other supportive measures such as oxygen therapy [2, 10]. At the time of writing this paper, biopharmaceutical companies such as Pfizer and Moderna have reported efficacy rates of 95 and 94.1% respectively for their vaccines, findings that show significant potential to protecting people from SARS-CoV-2 infection [11, 12].

As at November, 8, 2020, 50,407, 819 cases of COVID-19 have been recorded with 1,258,359 death and 35,639,301 recoveries worldwide (https://www.worldometers.info/). These indicate a case fatality rate (CFR) of 2.50% and case recovery rate (CRR) of 70.70%. In Africa, the number of cases, deaths and recoveries are 1,882,911; 44,936, and 1,584,617 respectively. Thus, the CFR and CRR of the continent are 2.39 and 84.16%. Whilst the effect on the African economy is clear, the lower cases recorded in Africa remains unclear. Following the introduction of vaccines in America and Europe, Africa as at 31/03/2021, had recorded 4, 108, 596 cases whilst Europe, America and Asia had recorded 41,506, 917; 54, 659,860 and 23, 298, 845 cases respectively. Of these, the reported deaths in Africa, Europe, America and Asia are 109, 944; 920, 952; 1, 312, 918 and 376, 820 respectively [13]. Although some experts attribute this to low testing and poor reporting in Africa, this might not be the exact case. Other factors such as genetics, social lifestyle, experience from managing previous outbreaks on the continent and strict safety protocols might be playing a significant role. Similarly; the role of sex disparity on COVID-19 remains poorly defined. Its unclear if estrogen could contribute to resistance in Africa especially in females given that women of African descent have higher estrogen levels and are also less infected compared to males. The review aims to decipher how the ovary could potentially contribute to the low CFR and high CRR recorded in Africa compared to the global values especially in females. This would help inform policy decision in the management of COVID-19.

## COVID-19 cases in Africa

Africa is a continent of approximately 1.3 billion people [14]. As reports of coronavirus (COVID-19) emerged from Wuhan, China, in December 2019 [15], African countries due to their close ties with China and other affected countries started anticipating the introduction of the virus to the continent. Health experts feared and projected a public health and economic catastrophe on the continent [16]. Projections were based on social lifestyle, weak health care systems, fragile infrastructure, inadequate availability of trained personnel, insufficient funding, inefficient data transmission as well as reduced access to medical supplies and equipment in the continent [14]. Interestingly, Africa is the last and least region the virus has affected as the pandemic spread across the globe [17]. Ivory Coast [18], in early January, 2020, followed by other African countries worked towards preventing COVID-19 importation and containing onward transmission within countries. These included surveillance at airports, closure of borders, quarantine and isolation packages, awareness campaign and imposition of curfews. All these contributed to the limited spread of the virus in African countries [19].

The first COVID-19 case in Africa was reported in Egypt on 14th February, 2020 [17, 20]. Chronologically, Egypt was followed by Algeria, with its first case reported on 25th February, 2020, followed by Nigeria on 27th of February, 2020 [21]. Most other African countries including South Africa, Ghana, Morocco, Algeria and Cameroon detected their first cases in March, 2020 [22]. Most of the initials cases were imported cases from Europe, which by 13th March, 2020 was the epicenter of COVID-19. This led to a surge in the number of cases in Africa and as of 18th April, 2020, 52 African countries had reported, 19,895 confirmed cases, while two countries (Comoros and Lesotho) were virus-free [20, 22]. However, by end of May, 2020, with the exception of Western Sahara, 54 of the 55 African Union Member States recorded a surge in coronavirus infection with approximately 100,000 cases reported [17]. At that point, most countries had experienced managing imported cases and community transmission. Cases of COVID-19 in Africa surpassed 200,000 by the second week in June and had escalated to 400,000 by 6th July [23]. Half of the 500,000 cases reported in the continent were from South Africa or Egypt [24]. In July 2020, the World Health organization voiced alarm at the spread of the pandemic in Africa stating that the surging numbers in South Africa could be a precursor for subsequent outbreaks across the continent [24]. Five countries had made up over 75% of the total confirmed cases which had exceeded a million by 6th August, 2020 [24]. These included South Africa, Egypt, Morocco, Ethiopia and Nigeria [24]. As of 3rd September, 2020, the continent had more than 1.2 million symptomatic cases [20].

The highest number of confirmed cases in the African continent as at November, 8, 2020, were detected in six countries; South Africa, Morocco, Egypt, Ethiopia, Tunisia and Libya with 734,175; 246,349; 108,754; 98,746; 66,334 and 66,444 cases respectively **(**Table1**)**. Contrary, the lowest number of cases were found in Sao Tome and Principe, Burundi, Comoros, Tanzania, Eritrea, Mauritius, Seychelles and Western Sahara with reported cases of 960, 606, 557, 509, 484, 453, 158 and 10 respectively **(**Table 1**)**. As of 8th November, 2020, the collective confirmed cases in Africa had reached 1,866,132 representing ~4.2% of the global total [25]. However, some experts challenge the true epidemiology of the pandemic as the exact case numbers are believed to be significantly higher than the confirmed counts and attribute this to inadequate testing capacity for COVID-19 in the continent. This might not be entirely true since African countries have shown pro-active commitments toward the containment of the virus by implementing lockdowns at the early stages of the pandemic, imposing strict safety protocols and establishing various testing centers [13, 26]. Therefore, it is inappropriate to attribute the low number of cases to poor testing capacity without taking into consideration genetic, social lifestyle, environmental and other adaptability factors.

**Table 1:** COVID-19 MORTALITY AND FATALITY IN AFRICA, WORLDOMETER (08/11/2020)

Country	Total Cases	Incidence Rate^**a**^	Total Deaths	Mortality Rate^**b**^	Fatality Rate^**c**^	Total Recoveries	Recovery Rate	Active Cases	Population
**Africa**	**1,866,132**	**19.1**	**44,961**	**3.3**	**2.4**	**1,563,497**	**83.8**	**257,674**	**1,351,785,936**
South Africa	734,175	65.2	19,749	33.2	2.7	675,593	92.0	38,833	59,573,601
Morocco	246,349	111.3	4127	11.1	1.7	200,954	81.6	41,268	37,065,690
Egypt	108,754	2.2	6343	6.2	5.8	100,106	92.0	2305	103,012,988
Ethiopia	98,746	33.7	1512	1.3	1.5	58,103	58.8	39,131	115,957,311
Tunisia	66,334	235.1	1721	14.5	2.6	36,727	55.4	27,886	11,862,542
Libya	66,444	389.7	915	13.3	1.4	38,624	58.1	26,905	6,904,355
Nigeria	63,731	1.3	1154	0.6	1.8	59,844	93.9	2733	207,922,463
Kenya	60,704	36.0	1093	2.0	1.8	40,131	66.1	19,480	54,186,063
Algeria	60,800	39.1	2024	4.6	3.3	41,510	68.3	17,266	44,129,712
Ghana	48,788	3.0	320	1.0	0.7	47,521	97.4	947	31,300,404
Cameroon	22,103	2.0	429	1.6	1.9	21,151	95.7	523	26,776,416
Ivory Coast	20,801	0.7	126	0.5	0.6	20,477	98.4	198	26,606,218
Madagascar	17,111	1.6	244	0.9	1.4	16,409	95.9	458	27,939,136
Zambia	16,819	3.3	349	1.9	2.1	15,862	94.3	608	18,562,871
Senegal	15,676	0.3	326	1.9	2.1	15,294	97.6	56	16,897,589
Uganda	13,852	13.0	131	0.3	0.9	7727	55.8	5994	46,240,646
Sudan	13,996	7.7	1115	2.5	8.0	9484	67.8	3397	44,207,179
Mozambique	13,485	6.7	99	0.3	0.7	11,275	83.6	2111	31,559,731
Namibia	13,143	56.0	133	5.2	1.0	11,578	88.1	1432	2,557,098
Guinea	12,363	11.6	73	0.6	0.6	10,751	87.0	1539	13,256,726
Angola	12,223	19.0	300	0.9	2.5	5626	46.0	6297	33,220,108
DRC	11,517	0.4	315	0.3	2.7	10,838	94.1	364	90,504,645
Cabo Verde	9224	136.4	100	17.9	1.1	8363	90.7	761	558,131
Gabon	9022	4.0	55	2.5	0.6	8878	98.4	89	2,244,088
Zimbabwe	8471	1.6	250	1.7	3.0	7983	94.2	238	14,939,395
Botswana	7835	96.0	27	1.1	0.3	5534	70.6	2274	2,368,293
Mauritania	7804	3.6	165	3.5	2.1	7469	95.7	170	4,692,224
Runion	6264	95.5	27	3.0	0.4	5380	85.9	857	897,590
Eswatini	5976	13.3	117	10.0	2.0	5704	95.4	155	1,164,431
Malawi	5942	2.1	184	1.0	3.1	5346	90.0	412	19,302,215
Djibouti	5604	6.2	61	6.1	1.1	5481	97.8	62	993,078
Congo	5379	25.2	92	1.7	1.7	3887	72.3	1400	5,565,493
Rwanda	5208	1.7	36	0.3	0.7	4953	95.1	219	13,064,256
Equatorial Guinea	5092	2.7	85	6.0	1.7	4968	97.6	39	1,418,901
CAR	4866	59.3	62	1.3	1.3	1924	39.5	2880	4,859,344
Mayotte	4550	560.2	45	16.4	1.0	2964	65.1	1541	275,114
Somalia	4229	5.5	107	0.7	2.5	3247	76.8	875	16,047,330
Gambia	3684	1.5	121	5.0	3.3	3527	95.7	36	2,440,259
Mali	3657	3.4	137	0.7	3.7	2817	77.0	703	20,453,317
South Sudan	2943	14.2	59	0.5	2.0	1290	43.8	1594	11,240,267
Benin	2745	1.9	43	0.4	1.6	2466	89.8	236	12,233,806
Burkina Faso	2562	0.6	67	0.3	2.6	2366	92.3	129	21,102,610
Togo	2460	8.2	57	0.7	2.3	1720	69.9	683	8,346,548
Guinea-Bissau	2414	25.7	42	2.1	1.7	1862	77.1	510	1,984,256
Sierra Leone	2373	6.1	74	0.9	3.1	1807	76.1	492	8,033,910
Lesotho	1967	41.8	44	2.0	2.2	1024	52.1	899	2,148,298
Chad	1538	0.5	99	0.6	6.4	1362	88.6	77	16,589,510
Liberia	1442	1.0	82	1.6	5.7	1310	90.8	50	5,099,231
Niger	1230	0.1	69	0.3	5.6	1143	92.9	18	24,507,853
Sao Tome and Principe	960	15.4	16	7.3	1.7	910	94.8	34	220,590
Burundi	606	0.8	1	0.0	0.2	511	84.3	94	12,013,565
Comoros	557	2.9	7	0.8	1.3	525	94.3	25	876,094
Tanzania	509	0.5	21	0.0	4.1	183	36.0	305	60,325,109
Eritrea	484	1.5	0	0.0	0.0	429	88.6	55	3,563,784
Mauritius	453	2.1	10	0.8	2.2	419	92.5	27	1,272,525
Seychelles	158	3.0	0	0.0	0.0	155	98.1	3	98,564
Western Sahara	10	0.2	1	0.2	10.0	0	0.0	1	602,465

^a^incidence rate per 100,000; ^b^mortality rate per 100,000 persons and ^c^fatality rate expressed in percentage

https://www.worldometers.info/Accessed on 8th November,2020

According to the Africa Centre for Disease Control and Prevention and the World health Organization, whilst other continents were dealing with a potential second wave, a slight increase in SARS-CoV-2 infections in Africa was recorded. As at 8th November 2020, Africa had recorded 252,718 active cases of COVID-19, with Morocco having the highest cases (42,708) followed by Ethiopia (38,386), South Africa (37,781), Libya, (27,069) Tunisia (21,143), Kenya (19,446) and Algeria (17,966) [27].

## COVID-19 interventions and recovery in Africa

COVID-19 case was first reported in Egypt on February, 2020, which makes Africa the last continent to be hit by COVID-19. With that, lessons were learnt from other continents on the pandemic, to act urgently on specific gaps and put in place stricter measures of detection, prevention, and control. Some of the strategic preventive measures deployed in Africa include complete and partial lockdowns, travel bans, closing of schools, companies, and offices, ban on large gatherings (including religious, sports, social and other events), systematic quarantines, increased testing capacity and strict infection control measures. The African task force for coronavirus (AFCOR) was established by Africa CDC to work with African Union Commission (AUC) and the WHO to manage the treatment of COVID-19 patients as well as propose interventions [28].

Other deployed measures included heightened surveillance and rapid identification of suspected cases through laboratory testing, patient transfer and isolation, contact tracing, and follow-up of potential contacts, regional coordination and funding, infection prevention and control (IPC), logistic mobilization and control such as PPEs, points of entry (POE) management, formation and deployment of rapid response teams (RRT), risk communication and community engagement (RCCE), expert training, mobilization and deployment [28]. The adoption and use of these variable technical and operational set of interventions is country specific. However, each country adheres to the WHO International Health Regulations (IHR) Monitoring and Evaluation Framework (MEF) [28, 29]. Efficient communication and timely dissemination of information through regional meetings and WHO developed platforms such as incident management system (IMS), Event Information System (EIS), Disease Outbreak News, and External Situational Reports have also played a key role in minimizing the devastating effects of COVID-19 in Africa [28, 29].

## Modern trends in laboratory testing of COVID-19 in Africa

Accurate testing of COVID-19 is a crucial step in controlling the spread of SARS-CoV-2. This has necessitated the use of highly sensitive and specific tests that could identify the virus at the earliest exposure. The testing methods deployed in Africa are the direct antigen detection and indirect antibody testing [30]. The direct antigen detection involves the direct identification of SARS-CoV-2 nucleic acid (RNA) and antigens in nasopharyngeal, oropharyngeal and sputum samples. RT-PCR and Xpert SARS-CoV-2 are the two (2) most widely used molecular testing methods in Africa; with the latter being a closed automated system which requires less sophisticated biosafety protocols. The choice of the methods is largely influenced by test sensitivity and specificity. A few serological tests have also been used as a rapid testing alternative in the absence of molecular PCR testing. These serological tests that meet the WHO criteria in terms of sensitivity and specificity have the advantage of short turnaround time, easy to perform with little training and logistics, large community testing and low cost of testing. The indirect testing involves the testing for antibodies in the blood of patients who have had prior infection.

A lot of African countries have advocated for the use of these test kits as it will provide a general idea of the level of exposure and perhaps the level of immunity in high risk groups. It has however, not been accepted by public health authorities mainly due to high level of false positivity. Africa is faced with the challenge of mobilizing enough resources for COVID-19 testing, thus, has adopted innovative ways of managing the huge COVID-19 testing burden. This involves pooling together samples from different individuals and testing them as if its one sample [31]. To ensure the quality and uniformity of protocols, Africa CDC developed guidelines and recommended 510 samples in a pool. However, the pooling efficiency is affected by sensitivity of RT-PCR assay, pool size and prevalence of COVID-19 within the population.

## COVID-19 fatality in Africa

The WHO defines COVID-19 deaths for surveillance purposes as any death which results from clinically compatible illness in an individual with probable or confirmed COVID-19 case, unless there is a clear evidence of an alternative cause of death unrelated to COVID-19 disease. Additionally, the individual should not have had the status of complete recovery from the time in between diagnosis and death. Globally, the COVID-19 related mortality rates may differ slightly mainly due to the source of data, differences in the inclusion and exclusion criteria as well as the time interval for reporting both cases and deaths by different countries. We present here data from the worldometer on COVID-19 **(**Table 1**)** (**https://www.worldometers.info/****)**. At the onset of the pandemic, many experts predicted millions of COVID-19 deaths in Africa mainly because of poor health systems, high illiteracy rates and poverty; however, Africa has recorded the lowest COVID-19 fatality. As of November 6, 2020, the African continent has recorded a total of 44,961 deaths as indicated by a lower case fatality rate (CFR) of 2.4% in comparison to the global CFR data of 2.6% **(**Table 1**)**. The low COVID-19 deaths in the African Region could be attributed to several reasons including Africa having a largely youthful population with more than 60% below 25years [32].

Comorbidities such as hypertension and diabetes contribute to the severity of COVID-19. In Gambia, although 6% of its populace are diagnosed with diabetes and 27% with hypertension, their mortality rate is lower compared to that of Europe and the Americas [33]. Other factors that may have influenced the low mortality in Africa include pre-existing immunity or exposure to similar infections and virulence of the viral strain, genetics, timely interventions, experience from managing previous pandemics and hormonal dynamics.

## Sex disparities in COVID-19 cases and mortality

Women are more likely to resist infectious diseases compared to men due to a perceived stronger immune system which are efficient in eliminating pathogens [34]. During the SARS-CoV and Middle East Respiratory Syndrome (MERS) pandemics, lower case-fatalities were observed in women compared to men [35]. This phenomenon has been replicated in the current COVID-19 pandemic where infection and mortality rates are relatively lower in women compared to men [9, 36,38]. It is also interesting to note that although women are generally at lower risk of COVID-19 infection, hospitalization and mortality, women in Africa have lower incidence and mortality rates compared to women in other parts of the world (https://globalhealth5050.org/).

**Fig. 1:**
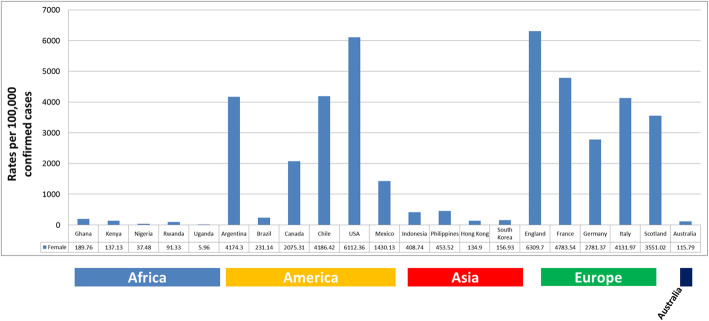
Female confirmed COVID-19 cases (rates per 100,000). Females from African countries have lower confirmed cases of COVID-19 compared to females in America, Asia, Europe and Australia

**Fig. 2:**
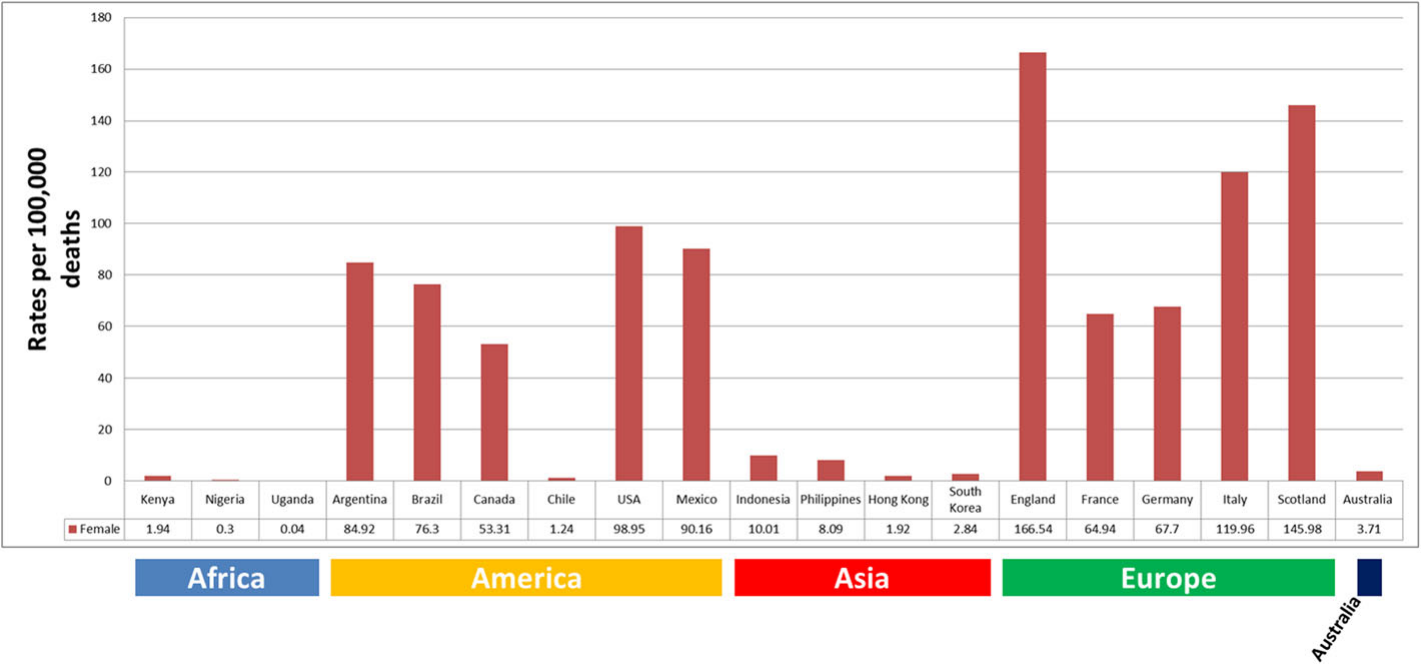
Female COVID-19 Deaths (rates per 100,000). Females from African countries have lower death rate of COVID-19 compared to females in America, Asia, Europe and Australia

In our interrogation of confirmed COVID-19 cases (rates per 100,000 women) between women across different continents using the https://globalhealth5050.org/ sex disaggregated data on COVID-19, we observed that African women had the lowest incidence rate **(**Fig.1**)**. Among African countries selected, Uganda had the lowest rates per 100,000 confirmed cases of 5.96 **(**Fig. 1**)**. On the average, women from Africa had rates per 100,000 confirmed cases of 92 compared to that of America (3035), Asia (289), Europe (4312) and Australia (116) **(**Fig. 1**)**. Using the available data on death cases reported by different countries into the database of https://globalhealth5050.org/, we compared the rates per 100,000 deaths of countries within our selected population **(**Fig.2**)**. Overall, women from Africa had the lowest mortality of 1.0 compared to America (67.0), Asia (6.0), Europe (113.0) and Australia (4.0) **(**Fig. 2**)**. Despite the poor economic status and health care systems in Africa, there have been an enviable incidence and low mortality rates especially within the female population. It is yet to be demonstrated why such disparities exist between women in Africa and those in the other parts of the world. Further investigation into this protective mechanism will provide helpful information in the management of COVID-19 worldwide, an approach that could be extended to other viral infections. We therefore hypothesize that the difference in lifestyles, age and the female reproductive endocrine dynamics provide an immune protection against COVID-19 infection and severity in African women.

## Racial differences in COVID-19 cases and mortality

**Fig. 3:**
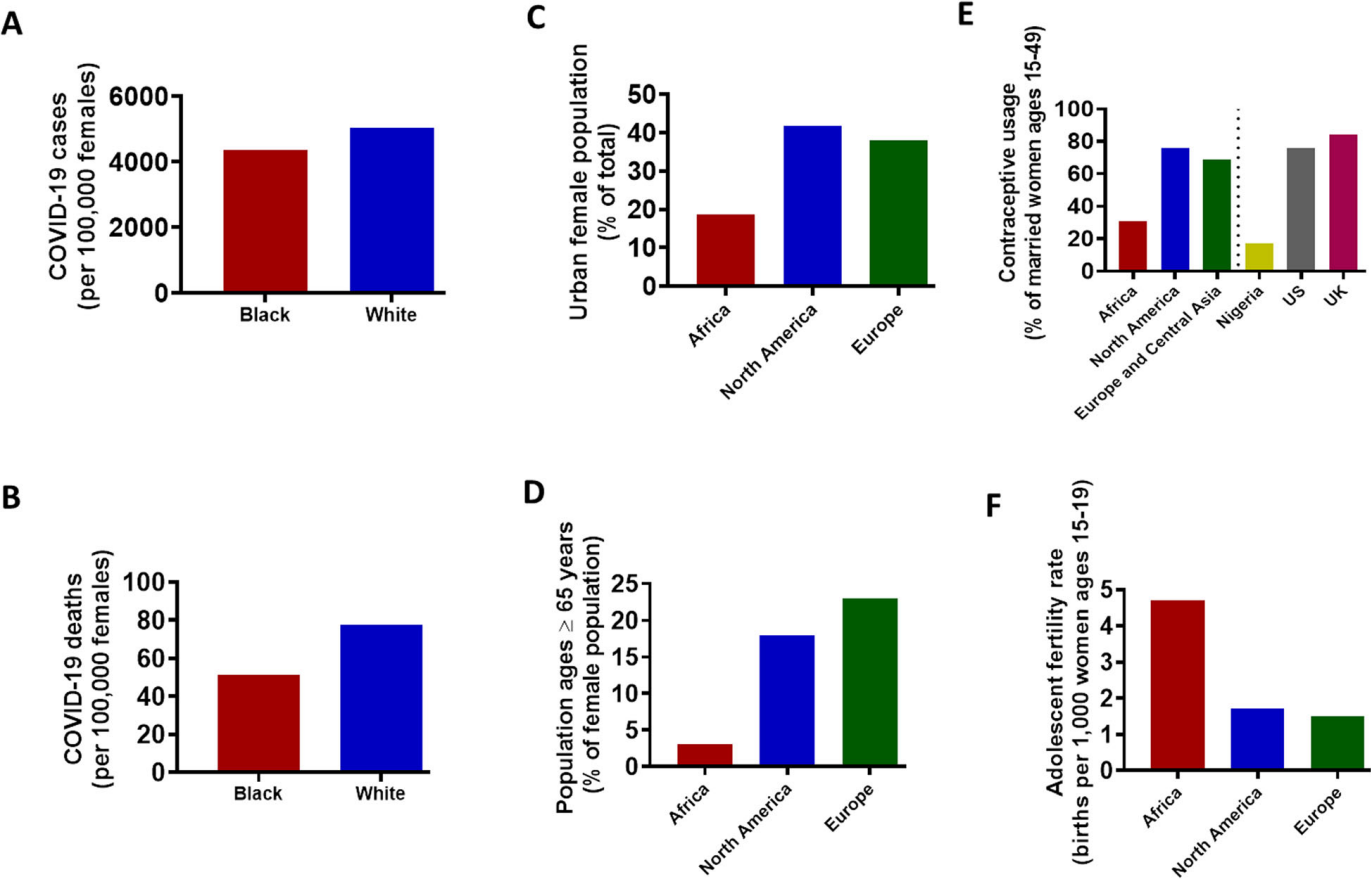
Sex, race, age, urban and contraceptive usage differences. **a** COVID-19 confirmed cases per 100,000 between non-Hispanic Black and White females in the US. **b** COVID-19 deaths per 100,000 between non-Hispanic Black and White females in the US. **c** Population of females in the urban areas. **d** Population of females ageS 65years and above. **e** Population of married women ages 1549years who have access to contraceptives. **f** Adolescent fertility rate calculated by births per 1000 women ages 1519years

Racial disparities to COVID-19 infection remains understudied. There is evidence on the role of race and ethnicity in COVID-19 infection. A number of studies conducted in the USA reported that Blacks were more susceptible to COVID-19 infection than their White counterparts. However, after adjusting for socio-demographic factors, comorbidities and age in some of the studies, people of African descent had slightly better prognosis [39,42]. In a study to assess the association of race and ethnicity with comorbidities and survival of COVID-19 patients at the Urban Medical Center in New York, non-Hispanic Black patients recorded a death rate of 17.2% whereas that of non-Hispanic patients was 20%. Additionally, a slightly improved survival was observed in the non-Hispanic Black population compared with their White counterparts [42]. In a COVID-19 race and ethnicity disparity study in the US as reported by the CDC, Black (non-Hispanics) females between the ages 1519years recorded 2915 cases per 100,000 while 4655 were recorded for Whites (non-Hispanics) females. For ages between 20 and 25years, 4316 cases per 100,000 were recorded for Black (non-Hispanics) females compared with 5867 for White (non-Hispanics) females (https://www.cdc.gov/mmwr/volumes/70/wr/mm7011e1.htm). Interrogating a CDC case-surveillance dataset (https://data.cdc.gov/Case-Surveillance/COVID-19-Case-Surveillance-Public-Use-Data-with-Ge/ynhu-f2s2) stratified according to race and sex, non-Hispanic White females recorded 5040 COVID-19 cases per 100,000 while their Black counterparts recorded 4364 **(**Fig.3a**)**. Also, 78 deaths per 100,000 were recorded for non-Hispanic White females as against 51 for non-Hispanic Black females **(**Fig. 3b**)**. However, the underlying mechanism for the sex, ethnic and racial disparities against COVID-19 remains to be studied. Although socio-economic and genetics factors have been implicated in racial/ethnic disparity against COVID-19, the protective role of estrogen and its activity have also been proposed as a potential contributing factor [43,45]. Given that non-Hispanic Black females have higher levels of bioavailable estrogen as well as increased estrogen activity compared with Whites females, its conceivable that estrogen dynamics might offer some explanations to the racial disparities in COVID-19 cases and mortalities [44, 46, 47].

## Racial differences in estrogen production

Estrogens are female reproductive hormones that are mainly produced by the ovaries, placenta and corpus luteum. Estrogen plays a key role in reproductive health, bone function, cardiovascular function and cognitive health [48]. In addition to that produced by the ovaries, a small but significant amount is also produced by the liver, brain, adipose tissue, skin and heart [48,50]. Estrogen is mainly produced in females; however, smaller levels are produced in males. Estrogen is present in three forms; namely, estrone (E1), estradiol (E2 or 17-estradiol) and estriol (E3). E1 is the weaker form of estrogen that is mostly present in post-menopausal women. E2 is mostly found in the reproductive ages of females and are the most common type of estrogen. E3 levels are elevated during pregnancy and crucial in maintaining the pregnancy. Estrogen is synthesized from androgen via the biosynthesis of an enzyme called aromatase. Aromatase is expressed primarily in the ovaries although some expression is also found in the placenta, skin, brain and adipose tissues [48,50]. The wide tissue expression of aromatase ensures that estrogen is produced in a required amount detectable in the blood for clinical diagnosis and prognosis of disease conditions.

With the decreased incidence, hospitalization and mortality rates amongst African women compared to non-African women **(**Figs.1 and 2**)**, we hypothesize that estrogen production dynamics could play a central role in the protection against COVID-19. Aromatase expression, estrogen levels and activity amongst women from diverse race and ethnicity have been analyzed in recent studies [47, 51,53]. Interestingly, estrogen levels as well as activity have been shown to be 35% higher in non-Latina black women compared to Latinas and non-Latina white women [47]. In a separate study, aromatase activity and estradiol levels were assessed between African-American women and Caucasian women across the menstrual cycle [51]. It was shown that although the African-American and Caucasian women were of similar age (27.2years) and body mass index (22.7kg/m^2^), estradiol levels were significantly elevated in the African-American women compared to their Caucasian counterparts [51]. The differences were more pronounced in the late follicular (225.214.4 vs. 191.510.2pg/ml; *P*<0.02), midluteal (211.922.2 vs.150.89.9, *P*<0.001) and late luteal (144.413.2 vs. 103.58.5, *P*<0.01) phases suggesting an increased aromatase activity in the African-American population [51]. Shaw et al. have also demonstrated that young African-American women have higher levels of ovarian aromatase mRNA expression, estradiol and decreased androgen to estrogen ratio compared to young Caucasian women [52]. These studies are consistent with others that have demonstrated increased levels of serum E1 and E2 in women of African descent compared to Caucasian women [53]. These suggest that the racial difference in aromatase expression could increase levels of estrogen production in women of African descent, an outcome that could explain in part why African-American women are associated with higher incidence of estrogen-responsive pathologies such as breast cancer, leiomyoma, increased bone density as well as early puberty age [44, 46].

Although racial differences could play a key role in estrogen levels, age, lifestyle, the use of contraceptives and pregnancy incidences could contribute to the differential estrogen levels observed. Rural and urban lifestyles in the selected geographic area could in part contribute to the differential estrogen production. Cardiovascular diseases highly correlate with increased sedentary and unhealthy lifestyles; behaviors that are common in the urban areas. Estrogen levels are inversely associated with LDL and triglycerides but positively correlates with high HDL synthesis, circulatory indicators used in the monitoring of cardiovascular diseases [54]. With only 18.6% of females residing in the urban areas of Africa compared with 41.7 and 38% in North America and Europe respectively (https://databank.worldbank.org/source/gender-statistics/Series/SP.URB.TOTL.FE.ZS) **(**Fig. 3c**)**, there is likelihood that urban activities could influence their estrogen production. The population of women in their premenopausal stages in Africa is considerably higher compared to their cohorts in the EU, America and Asia. Only 3% of females in Africa fall in the category of 65years and above compared to that of North America (18%) and Europe (23%) (https://data.worldbank.org/indicator/SP.POP.65UP.FE.ZS) **(**Fig. 3d**)**. With estrogen mostly produced in the reproductive age of females, this might contribute to the high levels of estrogen observed in women of Africa descent.

Contraceptive use amongst women with diverse ethnic backgrounds could potentially affect estrogen production thus, contributing to the differential levels. Contraceptives are known to directly act on ovaries resulting in a significant down-regulation of estrogen synthesis [55]. In Africa, only 31% of women ages 1549years have access to the use of contraceptives compared with North America (76%) and Europe and Central Asia combined (69%) (https://data.worldbank.org/indicator/SP.DYN.CONU.ZS) **(**Fig. 3e**)**. In Nigeria, only 17% within that age range have access to contraceptives compared with UK and US which are 84 and 76%, respectively (https://data.worldbank.org/indicator/SP.DYN.CONU.ZS) **(**Fig. 3e**)**. With only a small percentage of the reproductive women having access to contraceptives in Africa, this could in part explain why estrogen levels are higher in these population compared with their counterparts from other ethnic backgrounds. The use of contraceptives is directly reflected in the fertility rate recorded in each of the selected geographic areas. For instance, in Africa, 4.7 births per woman are recorded whereas 1.7 and 1.5 are recorded in North America and Europe respectively (https://data.worldbank.org/indicator/SP.DYN.TFRT.IN) **(**Fig. 3e**)**. Circulatory levels of estrogen are significantly high during pregnancy and this coupled with the high fertility rate observed in women of African descent, could potentially contribute to the reason why estrogen levels are high in these population [56]. The mortality rate of pregnant women infected with SARS-CoV-2 (0.16%) is significantly lower compared with that the American female population (2.24%) suggesting the potential immunological and protective functions of estrogen [57].

Despite the interesting correlation discussed above, its yet to be investigated if estrogen production is the central reason why lower incidence and case fatalities of COVID-19 are mostly seen in women of African descent. Here, we discuss the protective properties of estrogen that could potentially explain why women of African descent have lower incidence and case fatalities of COVID-19, information that could contribute to the fight against COVID-19.

## Potential anti-COVID-19 properties of estrogen

**Fig. 4:**
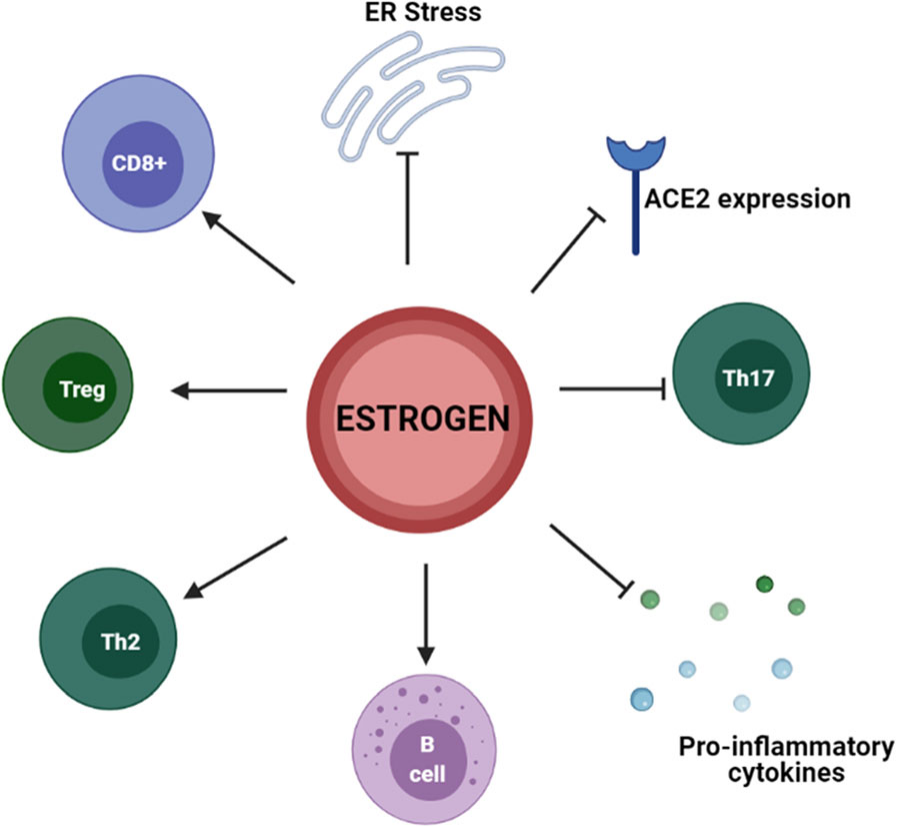
Anti-COVID-19 functions of estrogen. Estrogen production promotes Th2/Th1, activates T regulatory cells and CD8+ T cells. However, ACE2 expression, ER stress, pro-inflammatory cytokines and Th17 helper cells are inhibited by estrogen

COVID-19 severity is characterized by cytokine storm, decreased immune function, coagulation dysfunction, increased ER stress, increased expression of ACE2 as well as multiple organ dysfunctioning [5, 7, 58, 59]. Estrogen has anti-inflammatory function, reduce ER stress, improve immune cell functions and decrease the expression of ACE2 **(**Fig.4**)**. These suggest that increased levels of estrogen in African women could exert immune protection against COVID-19 infection, hospitalization and mortality, a potential reason for the low incidence, hospitalization and mortality rates.

### Anti-inflammatory function of estrogen

Estrogen plays a key role in the physiological functioning of the immune system. Estrogen receptors (ER) are expressed on CD4+ T cells, CD8+ T cells, B cells and monocytes suggesting a direct action of estrogen on these cells [60]. Increased levels of estrogen during experimental mouse and human studies resulted in decreased secretion of inflammatory mediators such as IL-1, IL-6, CCL2, intercellular adhesion molecule-1 (ICAM-1) and TNF- by inhibiting the NF-kB signaling pathway [61,63]. These cytokines especially IL-6 are primarily involved in COVID-19-related cytokine storm, a condition that is detrimental to the organs of the patients fueling poor patient outcomes [6, 64]. CCL2 is a chemo-attractant for the migration of neutrophils and monocytes to inflamed areas. Inhibiting their secretion thus, suppresses, alveolar edema, tissue-specific and systemic inflammation [63, 65]. E2 activates the signaling cascades in B cells leading to the secretion of antibody that helps in the fight against pathogens [61, 62]. Increased E2 production also promotes helper T cell type 2 (Th2): type 1 (Th1) leading to the secretion of anti-inflammatory mediators such as IL-10, IFN- and IL-4 [61, 62]. Although E2 suppresses the functions of Th17 helper cells (decreased secretion of pro-inflammatory IL-7), the functions of regulatory T cells (Tregs) are promoted to aid in immune tolerance [61, 63]. Despite the anti-inflammatory properties of estrogen, pro-inflammatory cytokines could overwhelm estrogen functions. TNF and IFN synergistically inhibit the expression of estrogen in granulosa cells (GC) [66]. Similar mechanism has also been demonstrated using lipopolysaccharides (LPS) [67]. IL-6 negatively regulates aromatase activity and estrogen production via MAKP signaling pathway in human granulosa tumor cell line (KGN cells) [68]. This suggests that the interplay of cytokines and estrogen are key to controlling the hyper-inflammation in disease conditions such as COVID-19. Cumulatively, these anti-inflammatory functions of estrogen could minimize COVID-19 severity in women, mechanisms that could partly explain why women of African descent are more resistant to COVID-19 complications as demonstrated by their lower mortality rate.

### Inhibition of ACE2 expression

ACE2 receptor is expressed by the lung cells as well as the upper respiratory tract and serves as an entry route for SARS-COV-2 infection. Thus, increased expression of this receptor is key to SARS-CoV-2 infection and subsequent COVID-19-related complications. Increased production of estrogen inhibits the expression of ACE2 receptors in bronchial epithelial cells, cardiocytes and kidney cells, a strategy that could help inhibit the entry and infection of SARS-CoV-2 [69]. Its therefore possible that the elevated levels of estrogen minimize SARS-CoV-2 entry hence the lower infection rate observed in African women.

### Suppression of endoplasmic reticulum (ER) stress

The endoplasmic reticulum (ER) is burdened with viral replication and protein translation (structural and nonstructural proteins of coronavirus) when the host cells are attacked by coronaviruses. These result in increased level of stress in the ER by forming glycosylation, double membrane vesicle (DMV) and depleting ER membrane lipids, activities that significantly affect the integrity of the ER [70]. SARS-CoV-2 infection of host cells produces similar ER stress that contributes to the severity of the disease. Estrogen production lowers ER stress by activating the unfolded protein response (UPR) signaling pathway [70]. This results in the restoration of the ER integrity in host cells as well as inhibits viral replication. Estrogen has been shown to suppress the replication and transmission of hepatitis B and influenza viruses minimize oxidative stress in cardiocytes [70]. Currently, community transmission of SARS-CoV-2 in Africa is minimal [71]. This could be explained in part by the elevated estrogen levels in African women, thus, suppressing viral replication and subsequently community transmission.

Females infected with SARS-CoV-2 virus are reported to have better prognosis compared with their male counterparts. However, they exhibit severe outcomes when infected with influenza viruses [38, 69, 70, 72,75]. Although SARS-CoV-2-mediated hyper-production of cytokines and chemokines in females result in clearing the viruses and improving patient survival faster than the males, a deleterious effect on pulmonary tissues are exhibited in females than males in situations of influenza infections [76,78]. Additionally, males produce increased levels of amphiregulin (AREG) compared to their female counterparts, a growth factor that promotes the repair of damaged tissues in the lungs as well as recovery [78, 79]. This suggests that host-mediated immunopathology plays a key role in influenza pathogenesis rather than viral titers, a condition that might explain why females are associated with severe influenza outcomes. Additionally, influenza pathogenesis significantly reduces ovarian function thus, inhibiting estrogen production [77, 80]; an effect that has not been implicated in SARS-CoV-2 infections. This suggests the relevance of estrogen production and activity in both SARS-CoV-2 and influenza viral infections.

### Estrogen as a potential hormonal therapy for COVID-19

To date, direct therapeutic options are limited to a modestly effective antiviral that remains inaccessible to most patients. Patients are managed using best supportive care including steroids, and in severe cases the use of mechanical ventilation and extracorporeal membrane oxygenation (ECMO) [81,84]. Identifying novel treatment modalities will enable physicians effectively manage COVID-19 patients effectively. One of the potential therapies being explored is estrogen since it has been shown to inhibit the production of pro-inflammatory cytokines, suppress the expression of ACE2 mRNA, stimulate antibody production, promote Th2/Th1 ratio and reduce ER stress [85]. In a retrospective study, the effects of systemic hormone administration (estradiol therapy) in women against COVID-19 death were analyzed [45]. Estradiol therapy significantly reduced the fatality risk for post-menopausal women by >50% lending to the strength that prospective studies on the potential protective role of estrogen should be investigated [45]. In a cohort study, women with COVID-19 receiving hormone replacement therapy (HRT) showed a higher survival rate supporting the protective effect of estrogen on COVID-19 [86]. Currently, clinical trials are ongoing to investigate the therapeutic efficiency of estrogen therapy in COVID-19 disease. These include estradiol patch (NCT04359329) and Norelgesetromin 6mg / Ethinyl estradiol 0.60mg (NCT04539626) which are being investigated in patients with COVID-19. In addition to estrogen, there are ongoing trials which are investigating other potential COVID-19 treatments such as progesterone (NCT04365127), enzalutamide (NCT04456049, NCT04475601), nafamostat (NCT04418128) and tamoxifen + isotretinoin (NCT04389580).

## Future directions and summary

COVID-19 is a major public health concern and efforts are being made to discover effective treatments, vaccines and biomarkers to efficiently manage it. The incidence and case fatality rates are significantly higher in males compared to women and this is mostly attributed to genetic, hormonal and immunological differences between men and women. Interestingly, women from African descent have lower COVID-19 incidence and mortality rates compared to non-African women. Given estrogen levels and activity are higher in women of African descent, its conceivable that this hormone might offer some protection against COVID-19 by suppressing greater production of cytokines, promoting anti-inflammatory cytokines, stimulating antibody production and suppressing ER stress **(**Fig.4**)**. We acknowledge that these suggestions are correlative hence a more mechanistic investigation is needed to substantiate this finding. Despite the promising findings discussed here, we also acknowledge the limitation of the number of sex-disaggregated data presented which are not reported by most countries. Thus, we limited our selection to countries that have all the parameters available. Also, we are not able to analyze the data based on age groups and total tests done since these parameters are missing in the dataset from most countries. Notwithstanding, the information presented here are compelling and warrant further investigation into why women of Africa descent have lower incidence rate, hospitalization and reduced mortality rates compared to non-African women. In summary, the greater production of estrogen in African women coupled with age, lifestyle, high fertility rate and contraceptive inaccessibility might be the contributing factors to resisting SARS-CoV-2 infection and minimizing COVID-19 severity.
